# Reduced serum 25(OH)D is closely related to bronchial mucus plug formation in children with mycoplasma pneumonia: A prospective cohort study

**DOI:** 10.3389/fpubh.2023.1099683

**Published:** 2023-01-26

**Authors:** Jiang Kun, Yan Silei, Chao Sun, Huang Wenyan

**Affiliations:** ^1^Department of Respiratory Medicine, Shanghai Children's Hospital, School of Medicine, Shanghai Jiao Tong University, Shanghai, China; ^2^Department of Nephrology, Shanghai Children's Hospital, School of Medicine, Shanghai Jiao Tong University, Shanghai, China

**Keywords:** *Mycoplasma pneumoniae*, mycoplasma pneumonia, particularly bronchial mucus plugs, 25 hydroxyvitamin D, children

## Abstract

**Background:**

The relationship between vitamin D nutritional status and the formation of bronchial mucus plugs (BMPs) is unclear. The aims of the current study were to investigate associations between serum 25(OH)D levels, serum inflammatory factors, and clinical characteristics in children with mycoplasma pneumonia (MPP), and to summarize the risk factors for BMPs in children with MPP.

**Methods:**

Clinical data from 175 children with MPP were collected and analyzed, the children were divided into a BMP group and a non-BMP group. Serum 25(OH)D levels, IL-8, and various inflammatory factors were compared in the two groups. Associations between 25(OH)D levels and IL-8, various inflammatory factors, and clinical characteristics were analyzed, and the diagnostic value of serum 25(OH)D levels was assessed.

**Results:**

Serum 25(OH)D level was significantly lower in the BMP group (*p* < 0.05). Serum IL-8 level, percentages of neutrophils, and some inflammatory factors were significantly higher in the BMP group (*p* < 0.05). Serum 25(OH)D level was negatively correlated with IL-8, neutrophil percentage, various inflammatory factors (all *p* < 0.05). It was also associated with lobular infection, pleural effusion, mechanical ventilation, and mycoplasma 2,063/2,064 mutation (all *p* < 0.05). In multivariate regression analysis 25(OH)D [odds ratio (OR) 0.98, 95% confidence interval (CI) 0.97–0.99, *p* = 0.003], IL-8 (OR 1.02, 95% CI 1.00–1.04, *p* = 0.002), polylobular infection (OR 1.75, 95% CI 1.17–2.64, *p* = 0.007), and MP DNA copies (OR 0.98, 95% CI 1.04–1.01, *p* = 0.022) were independent risk factors for BMPs, and the area under the curve value was 0.915 (95% CI 0.895–0.935). If the serum 25(OH)D level was <50 nmol/L, the respective percentages for sensitivity, specificity, positive predictive value, and negative predictive value were 97, 81, 78.9, and 97.6%.

**Conclusions:**

Vitamin D deficiency is common in children with MPP, and 25(OH)D levels are closely associated with inflammatory factors and disease severity in children. The serum 25 (OH) D level of MPP children with BMPs was lower than that of children without BMPs. Serum 25(OH)D can be used as a marker for the diagnosis of MPP in children with BMPs.

## Introduction

*Mycoplasma pneumoniae* (MP) can be detected in 8.0–66.4% of hospitalized children with community-acquired pneumonia, and it is one of the most common causes of that condition. MP can cause severe respiratory tract infections (1–3) that can result in different degrees of airway blockage by mucus plugs (4, 5). Bronchial shaping can develop if the airway obstruction cannot be relieved in a timely manner, and this can lead to bronchial inflammation stenosis, occlusion, granulation hyperplasia, and even legacy atelectasis, secondary bronchiectasis, occlusive bronchitis, and occlusive bronchiolitis sequelae. As a consequence, pulmonary function and the quality of life of children are substantially affected. It is therefore important for clinicians to rapidly determine whether bronchial mucus plugs (BMPs) have formed, because early diagnosis of BMPs is conducive to early symptomatic treatment and reducing the incidence of long-term complications (6, 7).

Vitamin D is an important regulator of calcium and phosphorus metabolism, and its role in maintaining calcium homeostasis and bone health has been widely recognized (8). Vitamin D has been studied in-depth, and its role in health and disease has attracted much interest from scholars. Many studies indicate that it has beneficial and regulatory effects on cardiovascular diseases, diabetes, obesity, cancer, and other diseases (9–12), and its biological functions also include anti-inflammatory and immunomodulatory effects (13, 14). Vitamin D deficiency can render children more prone to respiratory infections, including mycoplasma pneumonia (MPP) (15). Serum 25(OH)D level is negatively correlated with the severity of MPP, and low serum 25(OH)D may correspond with the severity of MPP (16).

Few studies have investigated relationships between vitamin D status and the clinical characteristics and progression of MPP. The relationship between vitamin D and the severity of MPP remains controversial. Whether 25(OH)D is involved in the formation of BMPs, and its relationship with BMPs is unknown. The current prospective cohort study is conducted to investigate associations between serum 25(OH)D levels and serum inflammatory factors and clinical characteristics in children with MPP, analyze risk factors in children with BMPs, and assess the diagnostic value of serum 25(OH)D levels with respect to BMPs.

## Patients and methods

### Patients

From March 2018 to March 2020, 175 children with MPP who were hospitalized and underwent bronchoscopic alveolar lavage at the Department of Respiratory Medicine of the Children's Hospital Affiliated with Shanghai Jiao Tong University School of Medicine were enrolled as research subjects. All children were in an acute stage of disease when they were admitted. MPP diagnostic criteria were serum MP-IgM titer ≥ 1:160 in the acute phase, or increased by four times or more in the recovery phase compared with the acute phase. Serum MP-IgM titers were measured *via* the passive agglutination method using a kit from Fujifilm Japonicum Co., Ltd. Mycoplasma was detected in bronchoalveolar lavage fluid *via* real-time fluorescent quantitative nucleic acid amplification (MP-qPCR) using a kit from Sun Yat-Sen University Da'an Gene Co., Ltd. Guardians of all the children provided written informed consents before bronchofibroscope examination, and the current study was approved by the hospital's Medical Ethics Committee (approval number 2018R164 E01).

The patients were divided into a BMP group (*n* = 73) and a non-BMP group (*n* = 102) depending on whether mucus thrombus blockage was detected *via* fiberoptic bronchoscopy. Under fiberoptic bronchoscopy the mucus secretions in the BMP group were found to be strips, blocking the respiratory tract (involving the lumen of one or more lung segments), and even in a plastic shape which could be removed by lavage and suction. In cases where they could not be easily removed, a cell brush or biopsy forceps were used. In the non-BMP group mucosal hyperemia, edema, longitudinal folds, and minor secretions were observed under fiberoptic bronchoscopy, which were thin and flocculent, without sputum thrombolysis, and could be easily removed *via* lavage.

Inclusion criteria were (1) age from 1 to 15 years; (2) meeting the diagnostic criteria for MPP; (3) clear and complete clinical records; (4) provision of informed consent; and (5) fiberoptic bronchoscopy completed. Exclusion criteria were (1) severe liver and kidney disease, osteoporosis, or thyroid disease; (2) a history of bronchiectasis or asthma; (3) congenital heart disease, congenital respiratory malformation, or congenital immune deficiency; (4) oral or intravenous use of glucocorticoids or immunomodulators in the past month, or vitamin D or calcium in the past 3 months; and (5) clear evidence of mixed bacterial, viral, or fungal infection.

### Data collection

Various data including age, sex, hypoxemia, shortness of breath, duration of fever, use of systemic corticosteroids, use of immunoglobulins, C-reactive protein (CRP), D-dimer, ferritin, lactate dehydrogenase (LDH), erythrocyte sedimentation rate (ESR), and alanine transaminase (ALT) were all extracted from medical records.

### Bronchofiberoptic alveolar lavage

Olympus fiberoptic bronchoscopes with different outer diameters were used depending on the age of the child, and local anesthesia was used to facilitate entry into the respiratory tract through the nose to observe lesions of the respiratory tract mucosa. After reaching the lesion site, secretions were collected for bacterial culture and drug sensitivity testing, then diseased lung segments were lavaged with 37°C 9 g/L saline (5–10 mL each time, 2–5 mL/kg). In cases in which the mucus plugs were difficult to remove *via* local lavage, brush or biopsy forceps were used to remove them. Upon completion of the procedure the fiberoptic bronchoscope was pulled out slowly.

### Inflammatory cytokine assays

Serum samples were collected from all patients within 24 h after admission, stored at −70°C, then subjected to inflammatory cytokine detection. Levels of 25(OH)D and IL-8 were tested using enzyme-linked immunosorbent assays.

### Statistical analysis

The most meaningful clinical features for diagnosing BMPs were identified *via* least absolute shrinkage and selection operator (LASSO) analysis. Univariate and multivariate logistic regression analyses were used to identify independent risk factors for BMPs. Statistical analysis was performed using R software (version 3.5.0). Enumeration data were expressed as numbers and percentages, and were compared using the chi-square test or Fisher's tests. Normally distributed data were represented by means ± the standard deviation, and non-normally distributed data were represented as medians and the interquartile range. Means and medians were compared using Student's *t*-test or Wilcoxon's rank test retrospectively. The data of both were normal distribution, and the correlation was determined by Pearson's correlation analysis, if one of the two was non-normal distribution data, the correlation was determined by Spearman's correlation analysis. *p* < 0.05 was deemed to indicate statistical significance.

## Results

### Baseline parameters in the BMP and non-BMP groups

Of the 175 children with MPP, 73 were assigned to the BMP group. Their ages ranged from 1 to 13 years, and they included 48 males and 25 females. There were 102 patients in the non-BMP group, their ages ranged from 1 to 14 years, and they included 63 males and 39 females. There were no significant differences in age or gender between the two groups (Table 1).

**Table 1:** Demographics and clinical characteristics of patients with MPP.

	**Total**	**Patients with** **BMPs (*n* = 73)**	**Patients with no** **BMPs (*n* = 102)**	***p*-value**
Gender, *n* (%)				0.635
Male	111	48 (65.8)	63 (61.8)	
Female	64	25 (34.2)	39 (38.2)	
Age (months)				0.293
Median	69.0	72.0	62.5	
IQR	41.0–85.0	47.5–93.5	40.0–85.0	
Length of symptoms (days)	11.9 ± 3.4	12.8 ± 4.1	11.3 ± 3.2	0.016
Length of fever (days)	9.7 ± 3.1	10.5 ± 3.3	8.8 ± 2.4	0.034
Short breath, *n* (%)				0.005
Yes	25	17 (23.3)	8 (7.8)	
No	150	56 (76.7)	94 (92.2)	
Mechanical ventilation, *n* (%)				0.005
Yes	14	11 (15.1)	3 (2.9)	
No	161	62 (84.9)	99 (97.1)	
Wheezing, *n* (%)				0.135
Yes	36	11 (15.1)	25 (24.5)	
No	139	62 (84.9)	77 (75.5)	
Respiratory tone, *n* (%)				0.013
Yes	36	22 (30.1)	14 (13.7)	
No	139	51 (69.9)	88 (86.3)	
Bubble, *n* (%)				0.017
Yes	110	38 (52.1)	72 (70.6)	
No	65	35 (47.9)	30 (29.4)	
Wheezing rale, *n* (%)				0.797
Yes	17	8 (11.0)	9 (8.8)	
No	158	65 (89.0)	93 (91.2)	
Pleural effusion, *n* (%)				<0.001
Yes	74	52 (71.2)	22 (21.6)	
No	101	21 (28.8)	80 (78.4)	
Polylobular infection, *n* (%)				<0.001
Yes	59	49 (67.1)	10 (9.8)	
No	116	24 (32.9)	92 (90.2)	
MP 2,063/2,064, *n* (%)				<0.001
Yes	79	55 (75.3)	24 (23.5)	
No	96	18 (24.7)	78 (76.5)	
Use of globulin, *n* (%)				<0.001
Yes	29	21 (28.8)	8 (7.8)	
No	146	52 (71.2)	94 (92.2)	
Use of glucocorticoids, *n* (%)				<0.001
Yes	84	47 (64.4)	37 (36.3)	
No	91	26 (35.6)	65 (63.7)	
MP DNA copies **(**10^4^/mL)				<0.001
Median	0.13	2.36	0.05	
IQR	0.05–1.75	0.05–6.13	0.05–0.27	
ESR (mm/h)				0.966
Median	48	48	48.5	
IQR	30–64	28.5–66.7	31.7–63.4	
D dimer (mg/L)				<0.001
Median	110	255	74.5	
IQR	61–299	115–634	53.8–124	
WBC count (10^9^/L)	9.4 ± 3.0	10.3 ± 2.9	8.7 ± 2.9	<0.001
Neutrophils (%)	61.9 ± 14.3	72.9 ± 8.9	54.1 ± 12.1	<0.001
CRP (mg/L)				0.009
Median	20	32	18.5	
IQR	9–45	8.5–68.5	9–33	
PCT (ng/mL)				<0.001
Median	29	48	19.5	
IQR	12–66	25–68.5	9–42.5	
LDH (U/L)				<0.001
Median	416	521	375	
IQR	323–598	379–858	297–478	
Albumin (g/L)	39.1 ± 5.6	37.3 ± 6.7	40.3 ± 4.3	<0.001
Ferritin (ng/mL)				<0.001
Median	143.7	283	113.6	
IQR	78.2–363.5	110–1,029	66.9–184.2	
ALT (U/L)				<0.001
Median	36	68	21	
IQR	18–63	48–84.5	12–36.3	
IL-8 (pg/mL)	55.1 ± 16.4	70.5 ± 9.3	44.2 ± 10.6	<0.001
25(OH)D (nmol/L)	52.9 ± 16.5	34.7 ± 7.1	66.1 ± 12.4	<0.001
Ig E				0.084
Median	117.3	143	89.2	
IQR	35.7–302	54.9–302	27.2–384.3	

MP, *Mycoplasma pneumoniae*; MPP, mycoplasma pneumonia; BMPs, bronchial mucus plugs; ESR, erythrocyte sedimentation rate; WBC, white blood cell; CRP, C-reactive protein; PCT, procalcitonin; LDH, lactate dehydrogenase; ALT, alanine transaminase; IL-8, interleukin-8; 25(OH)D, 25 hydroxyvitamin D; Ig E, immunoglobulin E; IQR, interquartile range.

### Serum 25(OH)D levels, clinical characteristics, and pro-inflammatory cytokines

**Figure 1:**
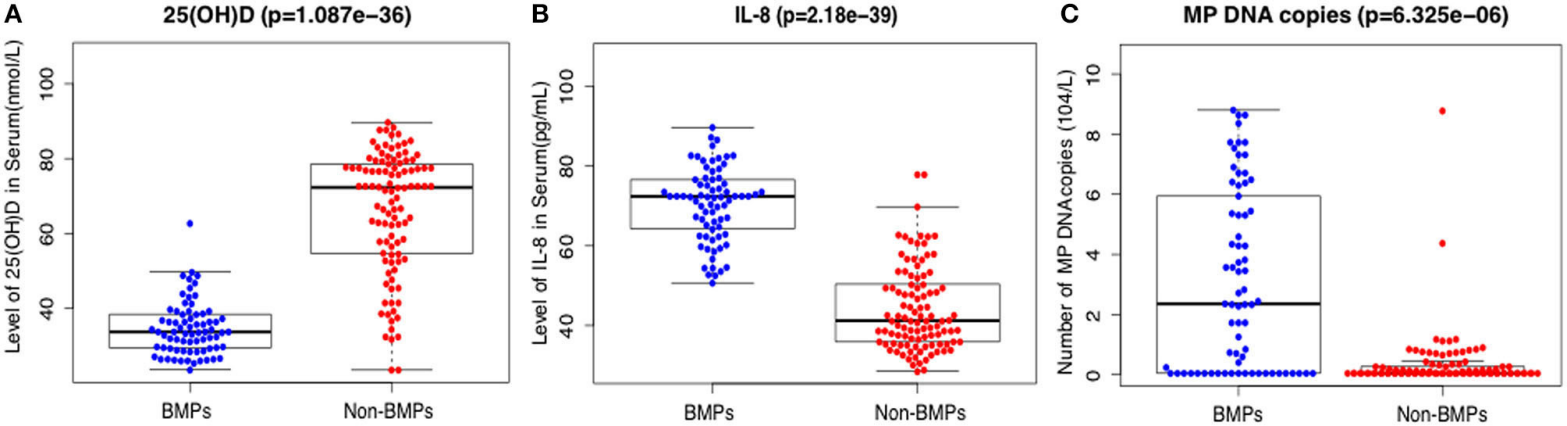
Comparison of serum levels of 25(OH)D **(A)**, IL-8 **(B)**, and number of MP DNA copies **(C)** in the BMP group and the non-BMP group.

The mean serum 25(OH)D levels were 34.7 ± 7.1 nmol/L in the BMP group and 66.1 ± 12.4 nmol/L in the non-BMP group, and the difference was significant (*t* = 15.2, *p* < 0.001) (Table 1; Figure 1A). D dimer, white blood cell (WBC) count, neutrophil percentage, CRP, PCT, LDH, ALT, albumin, ferritin, and IL-8 were all significantly higher in the BMP group (*p* < 0.05). There were no significant differences in ESR or IgE between the two groups (*p* > 0.05) (Table 1).

### Correlations between serum 25(OH)D and clinical characteristics

**Figure 2:**
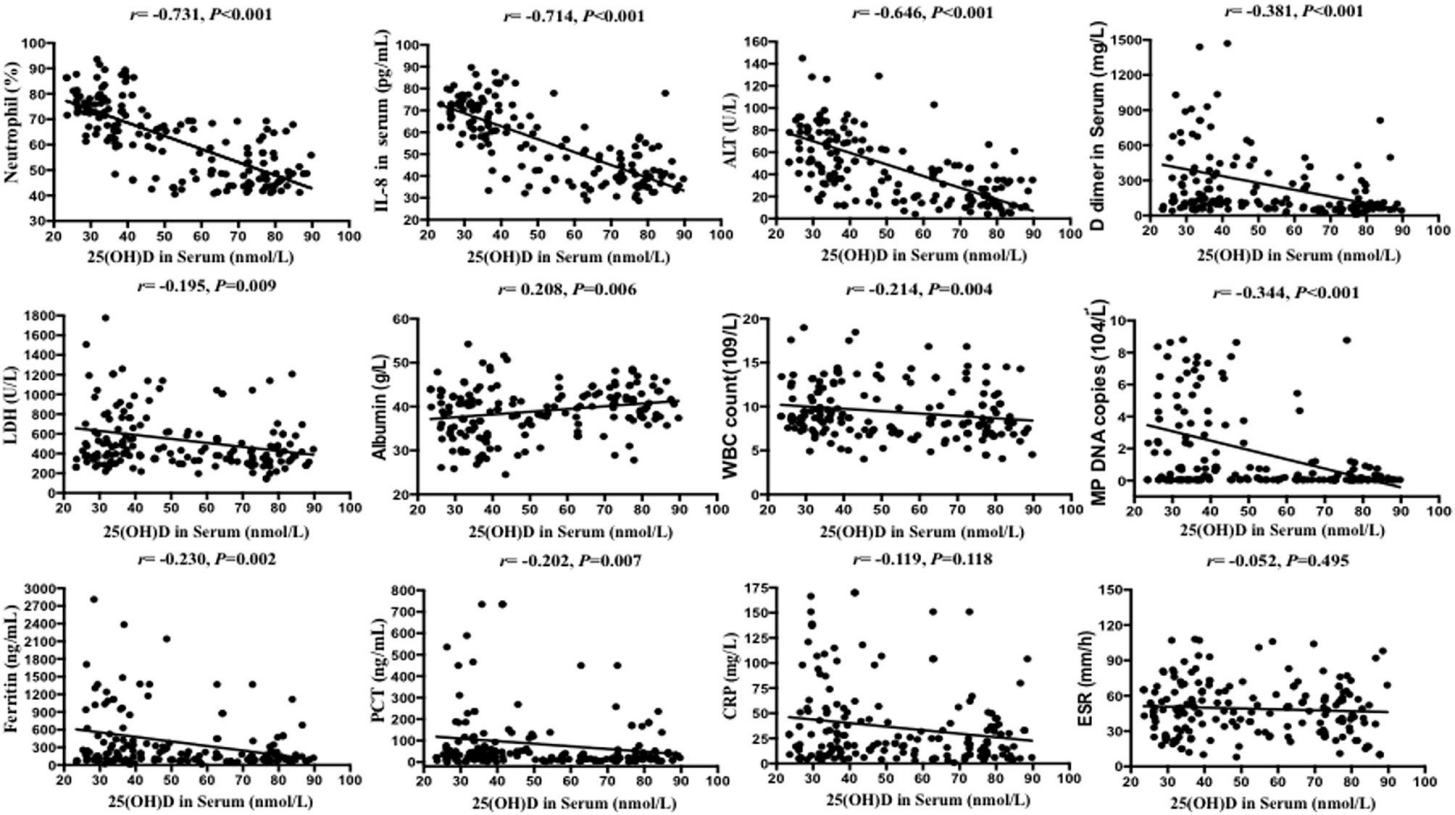
Scatter plots of Spearman's and Pearson's correlational analyses. Correlational analyses between 25(OH)D and neutrophils (%), IL-8, ALT, DD dimer, LDH, albumin, WBC count, MP DNA copies, ferritin, PCT, CRP, and ESR were performed. Each symbol represents the measurement of one case. The continuous line shows the least-square linear regression.

**Figure 3:**
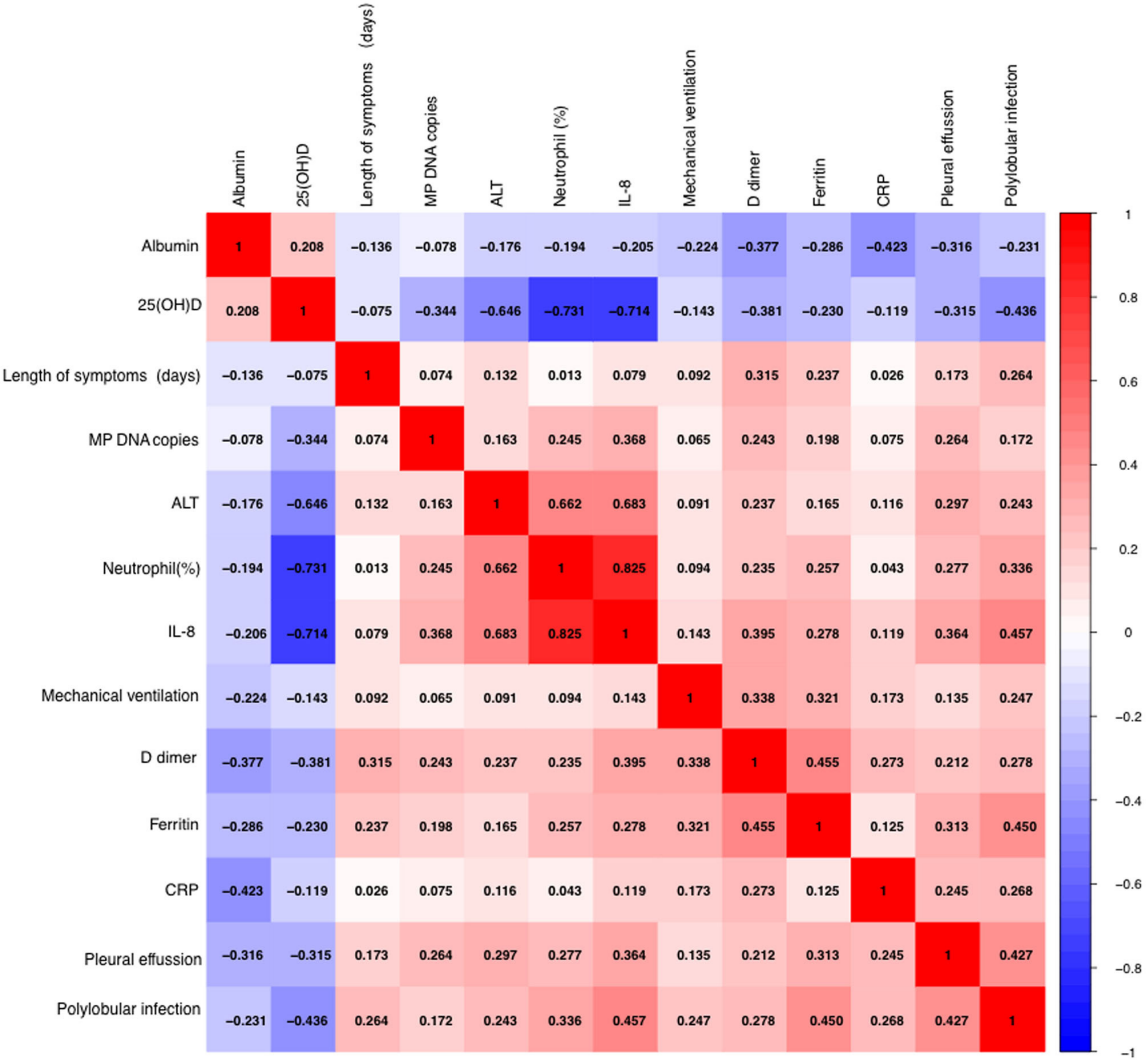
Spearman's and Pearson's correlational analyses of hematological and biochemical parameters. The correlation analysis matrix shows correlations between each of the 13 parameters. The color scale bar denotes correlation strength, with 1 indicating a positive correlation and −1 indicating a negative correlation.

**Figure 4:**
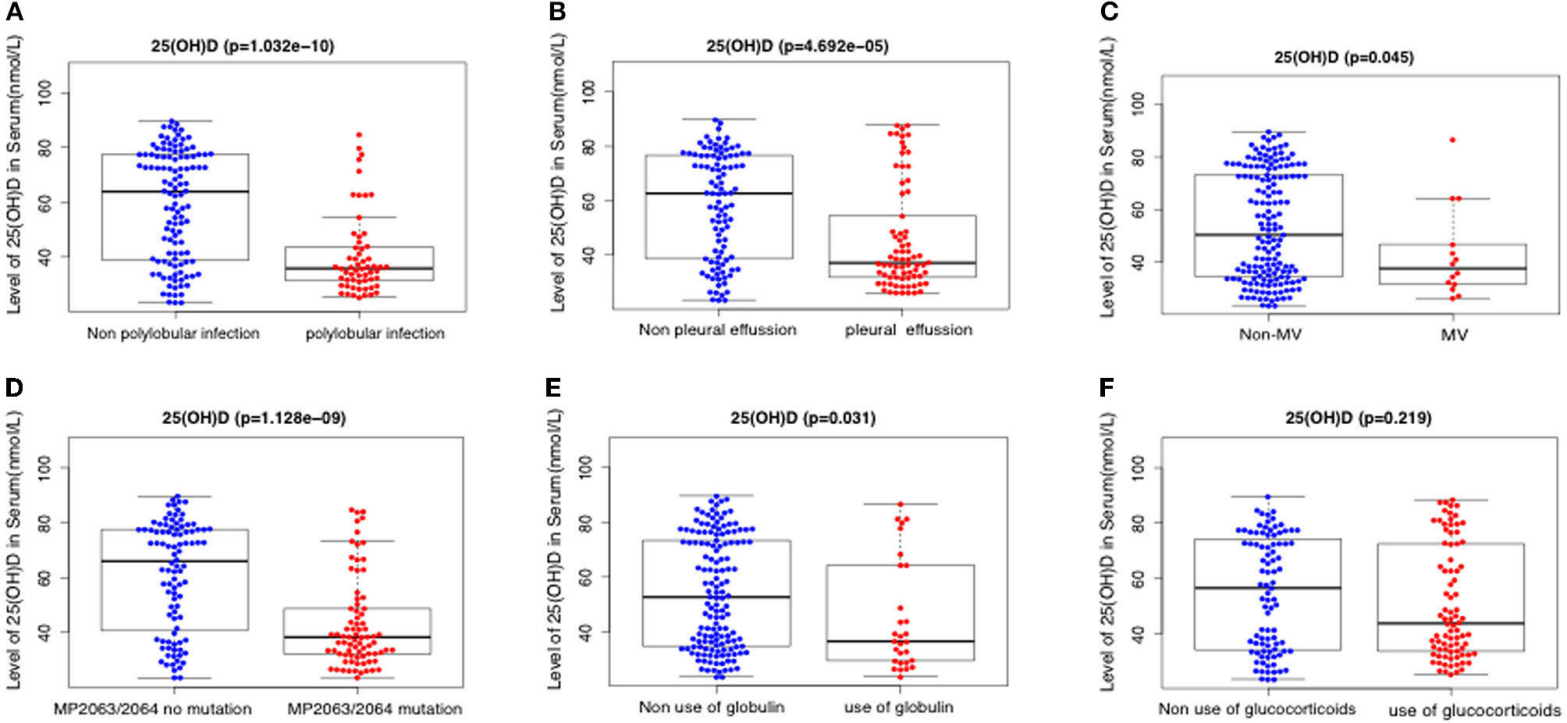
Relationship between serum levels of 25(OH)D and clinicopathological factors including polylobular infection **(A)**, pleural effusion **(B)**, mechanical ventilation **(C)**, MP 2063/2064 mutation **(D)**, use of globulin **(E)**, and use of glucocorticoids **(F)**.

Serum 25(OH)D level was negatively correlated with IL-8 level, neutrophil percentage, D dimer, ferritin, WBC count, LDH, ALT, PCT, and MP DNA copies, but positively correlated with albumin (all *p* < 0.05). There were no significant correlations between serum 25(OH)D level and CRP, length of symptoms, or ESR (all *p* > 0.05) (Table 2; Figures 2, 3). Serum 25(OH)D level was strongly associated with polylobular infection, pleural effusion, ventilator-assisted respiration, mycoplasma 2,063/2,064 mutation, and gamma globulin (all *p* < 0.05), but not with hydrocortisone (*p* = 0.219) (Figures 4A–F).

**Table 2:** Associations between serum 25(OH)D and clinical characteristics in children with MPP.

	***R*-square**	***F***	***r* (95% CI)**	***p*-value**
IL-8 (pg/mL)	0.542	204.6	−0.714 (−0.782, −0.630)	<0.001
Neutrophils (%)	0.548	209.6	−0.731 (−0.795, −0.650)	<0.001
D dimer (mg/L)	0.092	17.5	−0.381 (−0.504, −0.242)	<0.001
CRP (mg/L)	0.035	6.3	−0.119 (−0.266, 0.035)	0.118
Ferritin (ng/mL)	0.067	12.4	−0.230 (−0.370, −0.081)	0.002
Albumin (g/L)	0.052	9.5	0.208 (0.058, 0.350)	0.006
WBC count(10^9^/mL)	0.033	5.9	−0.214 (−0.355, −0.064)	0.004
Length of symptoms (days)	0.009	1.5	−0.075 (−0.225, 0.078)	0.322
LDH (U/L)	0.044	8.1	−0.195 (−0.337, −0.043)	0.009
ALT (U/L)	0.203	44.1	−0.646 (−0.727, −0.548)	<0.001
PCT (ng/mL)	0.029	5.3	−0.202 (−0.344, −0.051)	0.007
ESR (mm/h)	0.005	0.8	−0.052 (−0.203, 0.102)	0.495
MP DNA copies (10^4^/mL)	0.083	15.7	−0.344 (−0.472, −0.202)	<0.001

25(OH)D, 25 hydroxyvitamin D; MP, *Mycoplasma pneumoniae*; MPP, mycoplasma pneumonia; IL-8, interleukin-8; CRP, C-reactive protein; WBC, White blood cell; LDH, lactate dehydrogenase; ALT, alanine transaminase; PCT, procalcitonin; ESR, erythrocyte sedimentation rate.

### Biomarkers predicting the occurrence of BMPs

After applying LASSO analysis, 13 of the 29 candidate clinical parameters were further screened; 25(OH)D, length of symptoms, WBC count, D dimer, CRP, ferritin, albumin, mechanical ventilation, IL-8, ALT, pleural effusion, polylobular infection, and MP DNA copies (Supplementary Figure 1). In multivariate analysis 25(OH)D, IL-8, polylobular infection and MP DNA copies were independently associated with BMPs (Table 3). Serum IL-8 level and MP DNA copies were higher in the BMP group (Figures 1B, C).

**Table 3:** Logistic regression analysis of clinical characteristics associated with BMPs.

**Variable**	**Univariate analysis**			**Multivariate analysis**		
	**ß**	**OR (95% CI)**	***P***	**ß**	**OR (95% CI)**	***P***
Length of symptoms (days)	0.032	1.032 (1.006–1.075)	0.016	0.022	1.023 (0.982–1.065)	0.278
WBC count (10^9^/mL)	0.054	1.056 (1.007–1.106)	<0.001	0.051	1.052 (0.999–1.107)	0.051
D dimer (mg/L)	0.001	1.001 (1.001–1.001)	<0.001	0.0002	1.001 (0.999–1.001)	0.352
CRP (mg/L)	0.005	1.005 (1.001–1.008)	0.009	0.002	1.002 (0.997–1.006)	0.435
Ferritin (ng/mL)	0.001	1.001 (1.000–1.001)	<0.001	0.0001	1.001 (0.999–1.001)	0.464
Albumin (g/L)	−0.042	0.959 (0.930–0.989)	<0.001	−0.011	1.011 (0.977–1.046)	0.532
25 (OH)D (nmol/L)	−0.029	0.971 (0.963–0.979)	<0.001	−0.016	0.984 (0.974–0.995)	0.003
Mechanical ventilation	0.692	1.998 (1.153–3.463)	0.005	0.084	1.087 (0.579–2.039)	0.794
IL-8 (pg/mL)	0.045	1.046 (1.035–1.057)	<0.001	0.022	1.023 (1.008–1.037)	0.002
ALT (U/L)	0.006	1.006 (1.004–1.008)	<0.001	0.003	1.003 (0.999–1.006)	0.147
Pleural effusion	0.718	2.051 (1.513–2.781)	<0.001	0.315	1.369 (0.939–1.997)	0.102
Polylobular infection	1.071	2.918 (2.106–4.042)	<0.001	0.562	1.754 (1.166–2.637)	0.007
MP DNA copies (10^4^/ml)	0.062	1.064 (1.040–1.088)	<0.001	0.035	1.036 (1.005–1.068)	0.022

BMPs, bronchial mucus plugs; WBC, white blood cell; CRP, C-reactive protein; 25(OH)D, 25 hydroxyvitamin D; IL-8, interleukin-8; ALT, alanine transaminase; MP, *Mycoplasma pneumoniae*.

**Figure 5:**
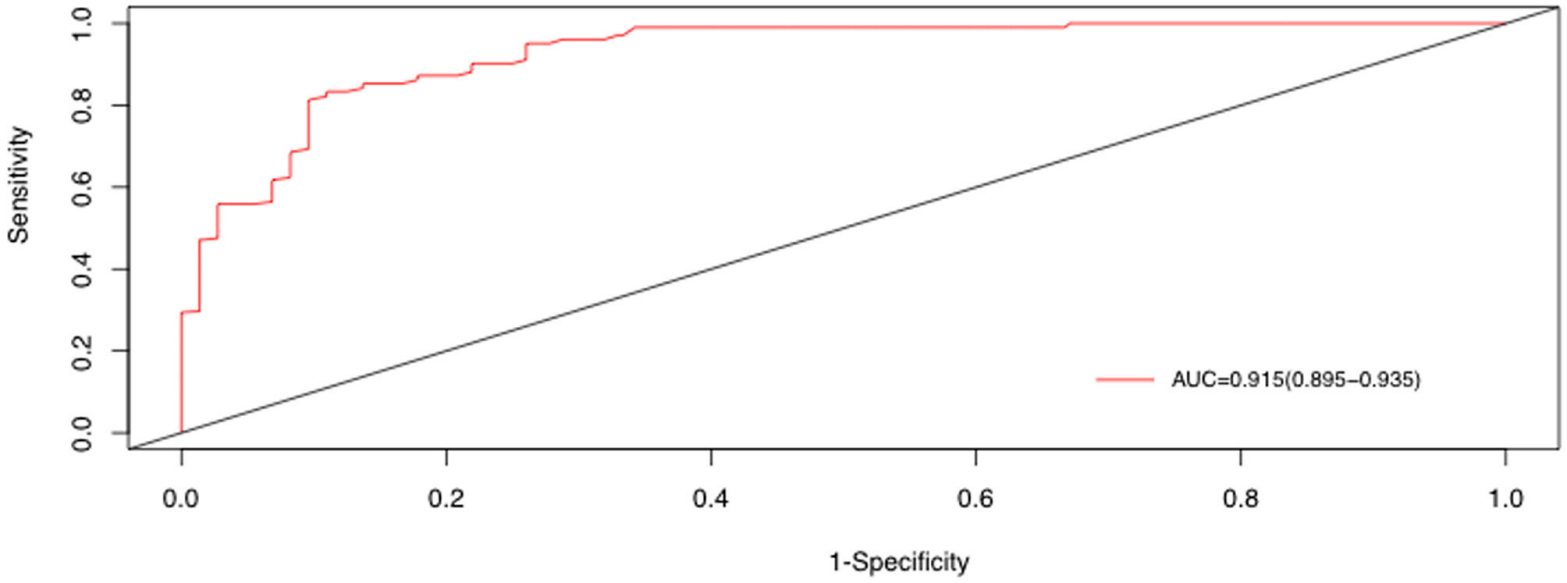
ROC curve for distinguishing BMPs, derived from the combination of 25(OH)D, IL-8, polylobular infection, and MP DNA copies.

Receiver operating characteristic (ROC) curves of 25(OH)D, IL-8, polylobular infection, and MP DNA copies were generated, and the best diagnostic values of these independent clinical points were obtained *via* cut point analysis (Supplementary Figures 2A–D). Combining the above four independent factors, the area under the curve (AUC) value was 0.915 [95% confidence interval (CI) 0.895–0.935] (Figure 5).

### Value of serum 25(OH)D in the diagnosis of BMPs

Serum 25(OH)D was significantly lower in the BMP group. A ROC curve based on serum 25(OH)D level indicated that the AUC value was 0.842 (95% CI 0.766–0.918). The cutoff value was 50.1 nmol/L (Supplementary Figure 2A). Seventy-one of the 73 children in the BMP group (97.3%) had a serum 25(OH)D level < 50 nmol/L. Nineteen of the 102 children in the non-BMP group (18.6%) had a serum 25(OH)D level < 50 nmol/L. When the serum 25(OH)D level was < 50 nmol/L the odds ratio of BMPs was 155 (95% CI 35–689), the positive predictive value was 78.9%, and the negative predictive value was 97.6%. The sensitivity was 97% (95% CI 89–99%), the specificity was 81% (95% CI 72–88%), the positive likelihood ratio was 5.2 (95% CI 3.5–7.9), and the negative likelihood ratio was 0.03 (95% CI 0.01–0.13) (Tables 4, 5).

**Table 4:** PPV and NPV of independent clinical characteristics for BMPs in children with MPP.

**Clinic features**	**BMP**	**Non-BMP**	**OR (95% CI)**	**PPV**	**NPV**	***p*-value**
25(OH)D (< 50 nmol/L)	71	19	155.1 (34.9–688.9)	0.789	0.976	<0.001
IL-8 (>58 pg/mL)	65	12	60.9 (23.6–157.5)	0.844	0.918	<0.001
Polylobular infection	49	10	18.7 (8.3–42.4)	0.831	0.793	<0.001
MP DNA copies (>1.2·10^4^/mL)	45	4	39.4 (13.1–118.9)	0.372	0.951	<0.001

PPV, positive predictive value; NPV, negative predictive value; BMPs, bronchial mucus plugs; MPP, mycoplasma pneumonia; 25(OH)D, 25 hydroxyvitamin D; IL-8, interleukin-8; MP, *Mycoplasma pneumoniae*.

**Table 5:** Sensitivity and specificity of independent clinical characteristics for BMPs in children with MPP.

**Clinic features**	**Sensitivity**	**Specificity**	**PLR**	**NLR**
25(OH)D (< 50 nmol/L)	0.97 (0.89–0.99)	0.81 (0.72–0.88)	5.22 (3.47–7.85)	0.03 (0.01–0.13)
IL-8 (>58 pg/mL)	0.89 (0.79–0.95)	0.98 (0.80–0.94)	7.57 (4.42–12.96)	0.12 (0.06–0.24)
Polylobular infection	0.67 (0.55–0.77)	0.90 (0.82–0.95)	6.85 (3.72–12.60)	0.36 (0.26–0.51)
MP DNA copies (>1.2·10^4^/mL)	0.62 (0.49–0.73)	0.96 (0.89–0.98)	15.72 (5.91–41.78)	0.39 (0.29–0.53)

BMPs, bronchial mucus plugs; MPP, mycoplasma pneumonia; PLR, positive likelihood ratio; NLR, a negative likelihood ratio; 25(OH)D, 25 hydroxyvitamin D; IL-8, interleukin-8; MP, *Mycoplasma pneumoniae*.

## Discussion

In the current study serum 25(OH)D levels were significantly lower in the BMP group than in the non-BMP group. Multivariate logistic regression analysis indicated that serum 25(OH)D level may be an independent predictor of BMPs. Meng et al. (16) reported that serum 25(OH)D levels in children in a severe mycoplasma infection (sMPP) group were significantly lower than those in a non-severe mycoplasma infection (nsMPP) group. Multivariate logistic regression analysis in that study indicated that serum 25(OH)D level could be used as an independent predictor of sMPP, which is closely related to the pathogenesis of MPP, and serum 25(OH)D level could be used as a diagnostic marker of sMPP. Those conclusions are consistent with observations in the present study. Notably however, Xing et al. (17) reported that although serum vitamin D levels in sMPP children were lower than those in nsMPP children, serum vitamin D level could not be used as an independent predictor of sMPP.

Mechanical ventilation, liver injury, and pleural effusion are the main indicators of severe condition in children with MPP (18). In the present study serum 25(OH)D level in children with MPP was significantly negatively correlated with several clinical characteristics, suggesting that low serum 25(OH)D level could be used as an indicator of the severity of disease in children with MP infection. In a growing number of studies there has been a significant association between vitamin D status and the incidence or severity of various infectious diseases in children, particularly upper and lower respiratory infections. In a large, multicenter, prospective cohort study of hospitalized children with bronchiolitis children with serum 25(OH)D levels < 20 ng/mL had a 2-fold greater risk of intensive care and 2-fold longer hospital stays (19). In a meta-analysis 25(OH)D deficiency increased the risk of mortality in children with sepsis and respiratory tract infection (odds ratio 1.81) (20). Brenner et al. (21) recently assessed associations between serum 25(OH)D levels and respiratory disease mortality over a 15-year follow-up period in a cohort of 9,548 adults, and reported a significant increase in respiratory mortality in vitamin D-deficient participants compared with non-vitamin D-deficient participants.

Early vitamin D supplementation may reduce and even prevent respiratory infections. Trivillin et al. (22) have suggested that early and regular oral vitamin D supplementation in infancy can effectively prevent respiratory tract infection. Some meta-analyses also indicate that vitamin D supplementation has a protective effect against respiratory tract infections in children, adolescents, and patients with severe vitamin D deficiency (23, 24).

Our results showed that the level of serum 25(OH)D was negatively correlated with IL-8, neutrophil percentage, ALT, LDH, D-dimer, ferritin, WBC count and PCT (all *p* < 0.05) in children with MPP. The absolute values of correlation coefficients of IL-8 and neutrophil percentage were >0.7, which indicated that 25(OH)D was strongly correlated with IL-8 and neutrophil percentage, and weakly correlated with other factors. Flores-Villalva et al. (25) found that the number of neutrophils in the experimental group (Spring born calves supplemented with commercial calcium containing pellets) was statistically significant decreased compared with the control group, which was consistent with our results. Some scholars have also shown that vitamin D3 supplementation in healthy people can reduce the serum level of IL-8, and its production was controlled by vitamin D through up-regulating the transcription of the anti-inflammatory gene DUSP1 (double specific phosphatase 1) (26, 27). We also found a significant positive correlation between the serum IL-8 level and the percentage of neutrophils (*r* = 0.825, *p* < 0.001). Neutrophils were recently described as producing extracellular traps to kill microbes, which would cause airway epithelial cells to secrete pro-inflammatory cytokine IL-8. Hudock et al. (28) showed *in vitro* that neutrophil extracellular traps selectively up-regulated the cytokine response of IL-1 family in bronchial epithelial cells, thereby enhanced the production of IL-8.

In recent years many scholars have discussed risk factors for BMPs. Zhao et al. (29) reported that peak body temperature, neutrophil ratio, platelet count, IL-6, and LDH were independent risk factors for BMPs. Xu et al. (30) retrospectively analyzed clinical data from 1,271 children with MPP and found that age > 5 years, IL-10 level > 10 ng/L, interferon gamma level > 30 ng/L, and the presence of complications were independent risk factors for BMPs. Therefore, the risk factors for BMPs are still controversial. In the current multivariate logistic regression analysis 25(OH)D level, IL-8 level, polylobular infection, and MP DNA copies were independent risk factors for BMP. The AUC value for diagnosing BMPs derived from the above four clinical factors combined was 0.915. In the present study 25(OH)D was identified as an independent diagnostic indicator for BMPs with a cutoff value of 50.1 nmol/L based on a ROC curve. If serum 25(OH)D level < 50 nmol/L was set as a cutoff value, the respective percentages for sensitivity, specificity, positive predictive value, and negative predictive value were 97, 81, 78.9, and 97.6%.

The current study had some limitations. The sample size was small, and all the children were from a single center. We did not consider the effects of seasonal changes on serum 25(OH)D levels. Due to time constraints the study lacked data on changes in serum 25(OH)D levels in children with MPP after vitamin D supplementation. Lastly, immune status and levels of inflammatory factors such as IL-4, IL-6, and TNF alpha were not determined.

## Conclusions

Vitamin D deficiency is common in children with MPP, and 25(OH)D level is closely related to inflammatory factors and disease severity in children. In MPP children, serum 25(OH)D levels are special lower in those with BMPs. Neutrophils and IL-8 may be involved in the formation of BMPs in children with MPP. Serum 25(OH)D can be used as a candidate marker for the diagnosis of BMPs in children with MPP.

## Data availability statement

The raw data supporting the conclusions of this article will be made available by the authors, without undue reservation.

## Ethics statement

The studies involving human participants were reviewed and approved by the Institutional Ethics Committee from Shanghai Children's Hospital (approval number 2018R164 E01). Written informed consent to participate in this study was provided by the participants' legal guardian/next of kin. Written informed consent was obtained from the individual(s), and minor(s)' legal guardian/next of kin, for the publication of any potentially identifiable images or data included in this article.

## Author contributions

JK conceptualized and designed the study and reviewed the manuscript. YS coordinated and supervised data collection and drafted the initial manuscript. CS collected the data and reviewed the manuscript for important intellectual content. HW conceptualized and designed the study and reviewed and revised the manuscript. All authors contributed to the article and approved the submitted version.

## Supplementary material

Supplementary Figure 1 **(A, B)** Variable selection using the least absolute shrinkage and selection operator binary logistic regression model.

Supplementary Figure 2 **(A–D)** ROC curves of different hematological and biochemical parameters, used to identify optimal cut-off values of 25(OH)D, IL-8, polylobular infection, and MP DNA copies for BMPs.

## References

[1] Kutty PK Jain S Taylor TH Bramley AM Diaz MH Ampofo K. Mycoplasma pneumoniae among children hospitalized with community-acquired pneumonia. Clin Infect Dis. (2019) 68:5–12. 10.1093/cid/ciy419 29788037 PMC6552676

[2] Chen J Yin Y Zhao L Zhang L Zhang J Yuan S. Mycoplasma pneumoniae infection prediction model for hospitalized community-acquired pneumonia children. Pediatr Pulmonol. (2021) 56:4020–8. 10.1002/ppul.25665 34547836

[3] Su M Wang Q Li D Wang LL Wang CY Wang JL. Prevalence and clinical characteristics of hospitalized children with community-acquired *Mycoplasma pneumoniae* pneumonia during 2017/2018, Chengde, China. Medicine. (2021) 100:e23786. 10.1097/MD.0000000000023786 33592835 PMC7870167

[4] Huang L Huang X Jiang W Zhang R Yan Y Huang L. Independent predictors for longer radiographic resolution in patients with refractory *Mycoplasma pneumoniae* pneumonia: a prospective cohort study. BMJ Open. (2018) 8:e023719. 10.1136/bmjopen-2018-023719 30567824 PMC6303577

[5] Brogan TV Finn LS Pyskaty DJ Jr Redding GJ Ricker D Inglis A. Plastic bronchitis in children: a case series and review of the medical literature. Pediatr Pulmonol. (2002) 34:482–7. 10.1002/ppul.10179 12422347

[6] Zhang J Wang T Li R Ji W Yan Y Sun Z. Prediction of risk factors of bronchial mucus plugs in children with *Mycoplasma pneumoniae* pneumonia. BMC Infect Dis. (2021) 21:67. 10.1186/s12879-021-05765-w 33441105 PMC7805118

[7] Huang JJ Yang XQ Zhuo ZQ Yuan L. Clinical characteristics of plastic bronchitis in children: a retrospective analysis of 43 cases. Respir Res. (2022) 23:51. 10.1186/s12931-022-01975-1 35248022 PMC8898471

[8] Chang SW Lee HC. Vitamin D and health - The missing vitamin in humans. Pediatr Neonatol. (2019) 60:237–44. 10.1016/j.pedneo.2019.04.007 31101452

[9] Cosentino N Campodonico J Milazzo V De Metrio M Brambilla M Camera M. Vitamin D and cardiovascular disease: current evidence and future perspectives. Nutrients. (2021) 13:3603. 10.3390/nu13103603 34684604 PMC8541123

[10] Pittas AG Jorde R Kawahara T Dawson-Hughes B. Vitamin D supplementation for prevention of type 2 diabetes mellitus: to D or not to D? J Clin Endocrinol Metab. (2020) 105:3721–33. 10.1210/clinem/dgaa594 32844212 PMC7571449

[11] Fiamenghi VI Mello ED. Vitamin D deficiency in children and adolescents with obesity: a meta-analysis. J Pediatr. (2021) 97:273–9. 10.1016/j.jped.2020.08.006 33022267 PMC9432231

[12] Negri M Gentile A de Angelis C Montò T Patalano R Colao A. Vitamin D-induced molecular mechanisms to potentiate cancer therapy and to reverse drug-resistance in cancer cells. Nutrients. (2020) 12:1798. 10.3390/nu12061798 32560347 PMC7353389

[13] Ao T Kikuta J Ishii M. The effects of vitamin D on immune system and inflammatory diseases. Biomolecules. (2021) 11:1624. 10.3390/biom11111624 34827621 PMC8615708

[14] Bernicke B Engelbogen N Klein K Franzenburg J Borzikowsky C Peters C. Analysis of the seasonal fluctuation of γδ T cells and its potential relation with vitamin D_3_. Cells. (2022) 11:1460. 10.3390/cells11091460 35563767 PMC9099506

[15] Yisak H Elmneh R Taklual W Ewunetei A Kefale B. Prevalence and associated factors of clinical vitamin A deficiency among preschool children 1–5 years of age in rural kebeles in farta district, South Gondar zone, Ethiopia: a mixed methods study. J Multidisciplin Health. (2020) 13:1191. 10.2147/JMDH.S279571 33116564 PMC7586053

[16] Meng F Chen P Guo X Li X Wu Y Liu W. Correlations between serum P2X7, vitamin A, 25-hydroxy vitamin D, and *Mycoplasma pneumoniae* Pneumonia. J Clin Lab Anal. (2021) 35:e23760. 10.1002/jcla.23760 33724522 PMC8128307

[17] Xing Y Sheng K Xiao X Li J Wei H Liu L. Vitamin A deficiency is associated with severe *Mycoplasma pneumoniae* pneumonia in children. Ann Transl Med. (2020) 8:120. 10.21037/atm.2020.02.33 32175413 PMC7049042

[18] Lee KL Lee CM Yang TL Yen TY Chang LY Chen JM. Severe *Mycoplasma pneumoniae* pneumonia requiring intensive care in children, 2010-2019. J Formos Med Assoc. (2021) 120:281–91. 10.1016/j.jfma.2020.08.018 32948415

[19] Vo P Koppel C Espinola JA Mansbach JM Celedón JC Hasegawa K. Vitamin D status at the time of hospitalization for bronchiolitis and its association with disease severity. J Pediatr. (2018) 203:416–22. 10.1016/j.jpeds.2018.07.097 30243543

[20] Cariolou M Cupp MA Evangelou E Tzoulaki I Berlanga-Taylor AJ. Importance of vitamin D in acute and critically ill children with subgroup analyses of sepsis and respiratory tract infections: a systematic review and meta-analysis. BMJ Open. (2019) 9:e027666. 10.1136/bmjopen-2018-027666 31122993 PMC6538078

[21] Brenner H Holleczek B Schöttker B. Vitamin D insufficiency and deficiency and mortality from respiratory diseases in a cohort of older adults: Potential for limiting the death toll during and beyond the COVID-19 pandemic? Nutrients. (2020) 12:2488. 10.3390/nu12082488 32824839 PMC7468980

[22] Trivillin A Zanella S Castaldo RJ Prati F Zanconato S Carraro S. Early oral nutritional supplements in the prevention of wheezing, asthma, and respiratory infections. Front Pediatr. (2022) 10:866868. 10.3389/fped.2022.866868 35402351 PMC8990313

[23] Jolliffe DA Camargo CA Jr Sluyter JD Aglipay M Aloia JF Ganmaa D. Vitamin D supplementation to prevent acute respiratory infections: a systematic review and meta-analysis of aggregate data from randomised controlled trials. Lancet Diabetes Endocrinol. (2021) 9:276–92. 10.1016/S2213-8587(21)00051-6 33798465

[24] Yang C Lu Y Wan M Xu D Yang X Yang L. Efficacy of high-dose vitamin D supplementation as an adjuvant treatment on pneumonia: systematic review and a meta-analysis of randomized controlled studies. Nutr Clin Pract. (2021) 36:368–84. 10.1002/ncp.10585 33037694

[25] Flores-Villalva S O'Brien MB Reid C Lacey S Gordon SV Nelson C. Low serum vitamin D concentrations in Spring-born dairy calves are associated with elevated peripheral leukocytes. Sci Rep. (2021) 11:18969. 10.1038/s41598-021-98343-8 34556723 PMC8460825

[26] AlGhamdi SA Enaibsi NN Alsufiani HM Alshaibi HF Khoja SO Carlberg C. Single oral Vitamin D3 bolus reduces inflammatory markers in healthy Saudi males. Int J Mol Sci. (2022) 23:11992. 10.3390/ijms231911992 36233290 PMC9569869

[27] Dauletbaev N Herscovitch K Das M Chen H Bernier J Matouk E. Down-regulation of IL-8 by high-dose vitamin D is specific to hyperinflammatory macrophages and involves mechanisms beyond up-regulation of DUSP1. Br J Pharmacol. (2015) 172:4757–71. 10.1111/bph.13249 26178144 PMC4594277

[28] Hudock KM Collins MS Imbrogno M Snowball J Kramer EL Brewington JJ. Neutrophil extracellular traps activate IL-8 and IL-1 expression in human bronchial epithelia. Am J Physiol Lung Cell Mol Physiol. (2020) 319:L137–47. 10.1152/ajplung.00144.2019 32159969 PMC7468846

[29] Zhao L Zhang T Cui X Zhao L Zheng J Ning J. Development and validation of a nomogram to predict plastic bronchitis in children with refractory *Mycoplasma pneumoniae* pneumonia. BMC Pulm Med. (2022) 22:253. 10.1186/s12890-022-02047-2 35761218 PMC9235233

[30] Xu X Li H Sheng Y Wu L Wang D Liu L. Nomogram for prediction of bronchial mucus plugs in children with *Mycoplasma pneumoniae* pneumonia. Sci Rep. (2020) 10:4579. 10.1038/s41598-020-61348-w 32165709 PMC7067858

