# Supplementary figures and images for: Reduced serum 25(OH)D is closely related to bronchial mucus plug formation in children with mycoplasma pneumonia: A prospective cohort study
Source: Front Public Health. 2023 Jan 26;11:1099683. doi: 10.3389/fpubh.2023.1099683 (PMC9909285; doi:10.3389/fpubh.2023.1099683)

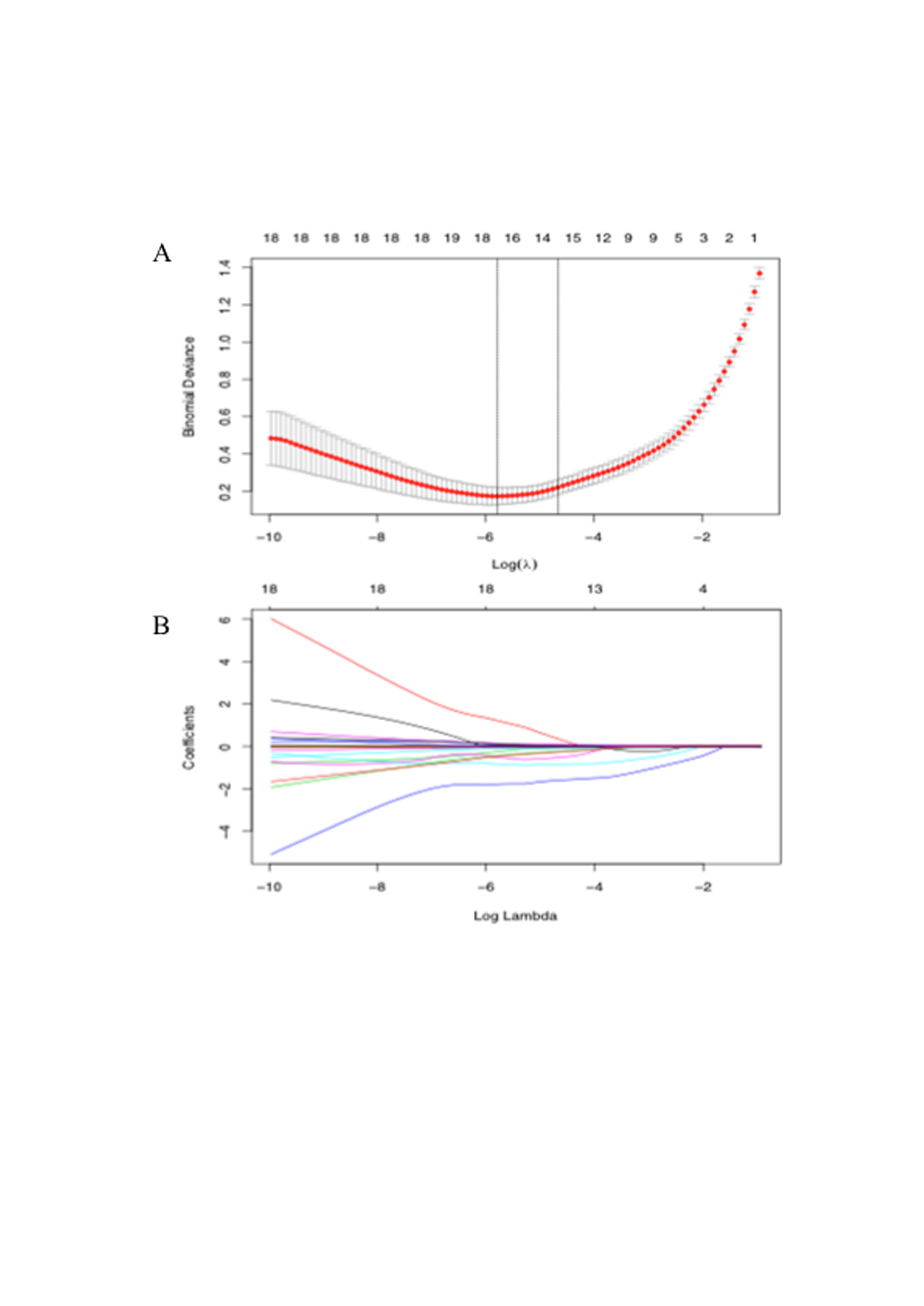

Supplement: Supplementary Figure 1 — (A, B) Variable selection using the least absolute shrinkage and selection operator binary logistic regression model. [file Image_1.tiff]

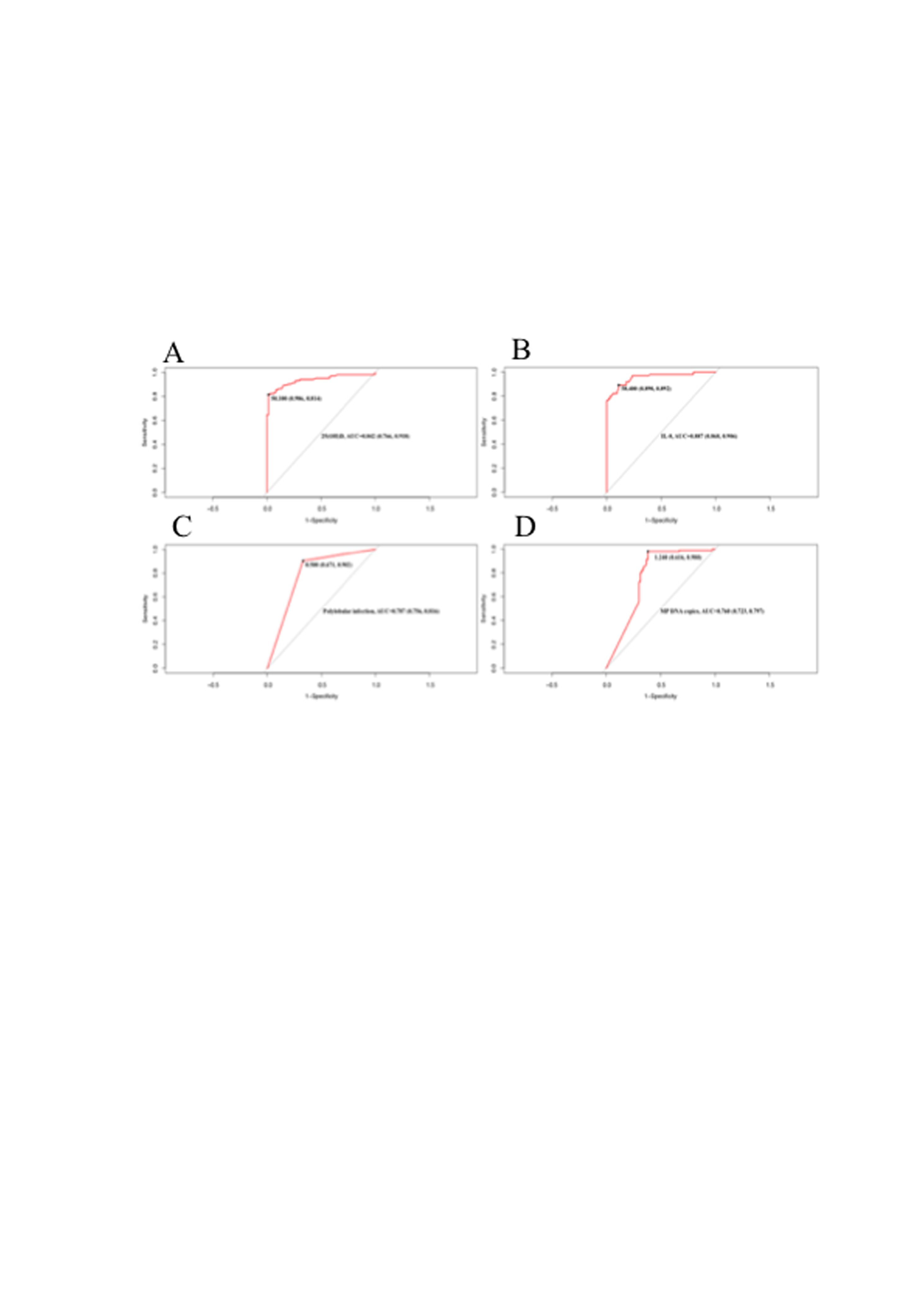

Supplement: Supplementary Figure 2 — (A–D) ROC curves of different hematological and biochemical parameters, used to identify optimal cut-off values of 25(OH)D, IL-8, polylobular infection, and MP DNA copies for BMPs. [file Image_2.tiff]
